# Make or break: Succeeding in transition from incarceration

**DOI:** 10.1371/journal.pone.0296947

**Published:** 2024-01-18

**Authors:** Heba Shahaed, Sai Surabi Thirugnanasampanthar, Dale Guenter

**Affiliations:** 1 Faculty of Health Sciences, McMaster University, Hamilton, Canada; 2 Faculty of Medicine, University of Toronto, Toronto, Canada; 3 Dalla Lana School of Public Health, University of Toronto, Toronto, Canada; 4 Department of Family Medicine, McMaster University, Hamilton, Canada; University of North Carolina at Chapel Hill, UNITED STATES

## Abstract

Several factors impact successful reintegration after incarceration. We sought to better understand these factors such as pre-release preparedness or access to financial resources in provincial correctional facilities in Ontario, Canada with an underlying focus on the role of personal identification (PID) among people at risk of homelessness. We conducted a qualitative study with one-on-one telephone interviews. Eligibility criteria included having been released from a provincial correctional facility in the preceding 2 years, being over the age of 18, speaking English and having telephone access. Participants were recruited between February 2021 and July 2021. All interviews were audio recorded and transcribed. Data was analyzed using a thematic analysis framework along with strategies from grounded theory research. We interviewed 12 individuals and identified six key themes including 1) Degree of Preparedness Pre-Release 2) Managing Priorities Post-Release 3) Impact of Support Post-Release 4) Obstacles with Accessing Services 5) Influence of Personal Identification 6) Emotions and Uncertainty. We found that people with mental health and addiction challenges are uniquely at risk post-release. Solutions must include comprehensive and proactive case management that bridges the pre-release and post-release periods, simplified processes for obtaining PID, better connections to health and social services, and improved pre-release planning for community support.

## Introduction

According to Statistics Canada, there are approximately 40,000 individuals in correctional facilities per day [1]. The poorer health status of this population when compared to the general Canadian population has been demonstrated through previous studies [2, 3]. Challenges with transitioning back to community living can contribute to poorer health for this population [3, 4]. The period post-release presents higher risk of mortality given challenges with issues such as employment, housing, medical conditions, substance use and mental health disorders [2, 3]. Additional challenges include systemic barriers to service use, experiences of discrimination from healthcare providers, inappropriate discharge planning, poor coordination of care with community services and a lack of personal identification (PID) [4–9]. Collectively, these factors make successful reintegration difficult and also contribute to the health disparities present among this population. Improving health for this population can be crucial to improving overall public health while also decreasing inequities [10, 11].

Challenges such as the stigma faced by individuals, difficulty with securing housing, a lack of financial resources and a lack of personal support have been explored in the literature in various contexts whether that be different countries or among a specific group such as female sex traffickers [12]. PID however has yet to be explored in the context of reintegration and specifically within Canada. A study conducted by Sanders et al. further, revealed that there currently exists a scarce amount of literature examining PID and its impact as a social determinant of health [13]. The limited literature reveals that a lack of government-issued identification can pose challenges in accessing healthcare in both the US and Canada [7, 9, 13]. Individuals are often released without appropriate identification such as a health card or birth certificate [9]. This is a problem as PID is often a requirement for access to various social and health services including opening a bank account, securing a residence, access to food banks, filling of prescriptions or access to health services [13]. Although single payer government health insurance is provided in Canada, in provinces such as Ontario a current health insurance card is usually required to be covered under the Ontario Health Insurance Plan (OHIP). The lack of a health insurance card can make receiving care especially difficult [7]. The process of obtaining PID is also particularly challenging for this population given the requirements of paying a fee, providing proof of citizenship, proof of residency, and at times a second form of PID [13]. For the 30 percent of individuals estimated to be homeless upon their release, retaining PID can also be difficult [14].

For this study, our intention was to explore the issues arising with reintegration among people released from incarceration, with particular interest in the role that PID plays among other challenges and priorities. We conducted in-depth interviews with individuals formerly incarcerated in provincial correctional facilities. Findings from this research will help inform transitional programs aimed to support reintegration along with providing data to drive key policy changes ultimately enhancing the health and well-being of this population.

## Methods

### Study design

This qualitative research study involved 30–60 minute semi-structured telephone interviews over a six-month period (February 2021 –July 2021). Both sampling principles drawn from literature which show that saturation is typically reached within the first twelve interviews [15, 16] and pragmatic considerations which involved availability of resources, funding constraints, and COVID-19 lockdowns, led us to conduct 12 individual interviews [15–17]. The Qualitative Research COREQ Checklist was also used to guide the methodology of this study to ensure thoroughness. All procedures performed involving human participants were in accordance with the ethical standards of the Hamilton Integrated Research Ethics Board under project number 12854.

### Study participants

Inclusion criteria for the study involved participants who were over the age of 18, spoke English, could provide informed consent, were released from a provincial jail (detention center, remand centre or correctional facility) within the past two years and had access to a telephone. Jails and detention centers are designed to hold individuals who may be on remand, have shorter sentences (60 days or less), or are waiting to be transferred to a correctional facility [18]. Correctional facilities hold individuals who serve longer sentences and also provide access to education and work programs [18]. As experiences at provincial and federal facilities can greatly vary, only individuals released from provincial facilities were included [19].

Given the restrictions in-place due to COVID-19, all interviews were conducted over the phone. Participants who did not have a phone were connected with community organizations who were able to provide them with access to a phone and a private space to allow the interview to be conducted. Participants were recruited primarily from Hamilton, Ontario through the support of local community organizations and posters. This was given the location of the investigators and supporting community organizations. The challenges of participant recruitment during COVID-19 encouraged us to employ convenience sampling. The pandemic also resulted in Hamilton correctional facilities relocating female inmates to a distant location which impacted the population of participants recruited. Participants were compensated for their time through a $20 gift card.

### Data collection

Researcher HS, an undergraduate student who was self-trained in interview facilitation while also being coached by DG, a family physician with abundant experience in qualitative research, made initial contact with participants. HS was responsible for conducting interviews with participants. These interviews were semi-structured as they followed an interview guide which focused on areas related to the participants experience prior to being released, during release and after release with an underlying focus on how PID played a role throughout these experiences. No one else beyond HS and the study participant were present during the telephone interview. HS was also responsible for obtaining informed verbal consent, audio recorded all interviews, took electronic field notes, and transcribed interviews after completion. Through recognizing that the population being studied could face challenges with having access to electronic resources where they may be able to review consent forms and send them back to researchers, an oral consent process was selected. An oral consent script was developed through adapting the template provided by the Hamilton Integrated Research Ethics Board and was used to provide specific and accurate information to participants about the study. A participant log was also maintained highlighting a participant’s ID and the date on which they provided oral consent. Each participant’s audio recording also provides evidence of their verbal consent. No prior relationship existed with study participants and information on personal goals for conducting the research or prior assumptions/ideas were not disclosed. Transcripts were not returned to participants for comment and/or correction and they did not provide any feedback on findings.

### Data analysis

We analyzed data using a thematic analysis framework developed by Braun and Clarke along with strategies from grounded theory research described by Charmaz [20, 21]. We employed the six phases of thematic analysis including familiarizing ourselves with the data, generating initial codes, searching for themes, reviewing themes, defining and naming themes, and producing the report. We began with data transcription where we became well acquainted with the data and the voice of our participants. This was followed by code generation that occurred as we had two independent reviewers coding each transcript and frequently discussed similarities and differences. NVIVO 12 was used to support the coding process. While data was inductively coded, strategies from grounded theory including initial coding and focused coding were also employed, ensuring coding was systematic and comprehensive. Codes were then used to generate appropriate themes that captured the data. Themes were frequently reviewed as they were constantly compared to each other, codes and the data overall. The six key themes that formed the structure of our coding tree include 1) Degree of Preparedness Pre-Release 2) Managing Priorities Post-Release 3) Impact of Support Post-Release 4) Obstacles with Accessing Services 5) Influence of Personal Identification 6) Emotions and Uncertainty. Through continued analysis and discussion, themes were named and defined enabling the production of a report. With an initial target of 12 interviews, we found during our analysis that data saturation had been reached after we had completed all 12 interviews as no new themes were observed in the data and no new codes were being generated [15].

## Results

### Participants

We conducted 12 in-depth semi-structured interviews. Participant characteristics are identified in Table 1 below.

**Table 1:** Participant characteristics.

Age	3 (18–29)	9 (30–60)	
Gender	9 Male	2 Female	1 Transgender Female
Ethnicities	8 White	3 Indigenous	1 Filipino
Housing	11 Street/Shelter/Transient Accommodation	1 Secure Long-Term Housing	
Previous Incarceration	1 No Prior Incarcerations before Current Experience	11 Previously Incarcerated	

### Themes

Interviews revealed six key themes: 1) Degree of Preparedness Pre-Release 2) Managing Priorities Post-Release 3) Impact of Support Post-Release 4) Obstacles with Accessing Services 5) Influence of Personal Identification 6) Emotions and Uncertainty.

#### Degree of preparedness pre-release

Events occurring prior to release influenced inmates’ reintegration experiences. When the timing of release was unexpected, there was no opportunity to prepare:*”…all of a sudden thrown into a situation where they all of a sudden they withdraw charges and you’re just dropped on the street and nothing*, *absolutely nothing*.*”* Returning to activities that originally led to arrest was sometimes the result. As one participant described,

*“so if a woman gets released with nothing*, *the first thing you’re thinking*, *she says I’m hungry I’m going to go get a chocolate bar*, *you know what I mean so right away you go over to the facilities across the way like the town and just start stealing*.*”*

The social worker in the institution was sometimes accessible and helpful. “*So this social worker is really proactive there um…since I was on a mental health block…like…um…she would come up to the floor almost daily and see if anybody needed anything*, *had any concerns*.*”* Other times access to this kind of help was difficult, or involved a long wait.

*“I put in like multiple requests to see this*, *to talk to a social worker just for like a lot of things like I wanted to email someone and like let them know something and a whole bunch of other things that they were allowed to do but then I never even seen them*.*”*

Participants also described that the programs and resources that they did have access to *“weren’t adequate enough I don’t think*. *They’re weird*, *they’re there but they’re not*, *you know what I mean*.*”* Medications prescribed during incarceration were also not always considered by the institution on release. Medical, mental health and addiction issues would then be suddenly uncontrolled. When planning was not carried out for continuation of medication on release, mental and physical health and addiction issues would become unstable.

*“I said you got the meds that I’m supposed to have for today*, *after that obviously I’m not getting any*. *She says well I’m going to the back […] she prepped a three day what do you call it release of for pills and then I got those for to last me for morning and night for three days and that was it*.*”*

#### Managing priorities post-release

Priorities for organizing life on release were often top of mind. These included securing housing, taking care of personal health, following up with arrangements such as those made by a social worker, securing finances and basic necessities, obtaining PID, avoiding old habits, and meeting court-ordered release conditions. One participant described *“a little responsible list of things I knew I wanted to take care of immediately*,*”*

There appeared to be a hierarchy among priorities, but not the same for everyone. For some, there was urgency for housing. One participant shared how they were worried about *“…where I’m going to go and where I’m going to live after leaving the shelter* …*I’m in an overflow shelter so […] it’s supposed to be very temporary so like now it’s kind of only staying focused on housing*.*”*

Approaches to managing health varied, with some discussing urgency to connect with a family doctor to arrange for their medications or ongoing health concerns while others talked about maintaining their sobriety and managing their mental health. Receiving opioid agonist therapy in a timely manner was particularly emphasized amongst participants. Another common priority for many was following up on the arrangements that were made for them in jail or post-release.

*“The first thing that I had to do was get in contact with the treatment centre because that was like a requirement of me being released*. *[…] I was in a rush to do that because the quicker I finished that program*, *the quicker I could go into like my employment training program and like start getting paid from that*. *[…] Um also*, *had to contact…like I was on the health care connect list already but I didn’t know how long that would take so I was also really worried about finding a doctor right away because I was only released with like so much medication and stuff*.*”*

Securing finances involved arranging for income through work, welfare or disability payments, and opening a bank account to keep funds safe. The need for more funds and limited available resources was echoed by multiple participants.

*“I got no way to keep cash pretty much you know what I mean […] I mean 600 bucks for a hole in the wall and then you’ve got $20 left over […] you buy a phone card $50*, *buy five packs of cigarettes that’s $50 and you’re lucky to spend $60 on food*, *you know what I mean $40 on food to be realistic and then you got $60 for social money”*

Other important priorities included obtaining PID as a participant had talked about how *“one of the first stops I made was Service Ontario to get my health card replaced and photo ID card sent out […] It’s one of the first things I have to take care of*.*”*

Trying to *“stay away from old acquaintances”* could be part of a strategy to live a new life. We noted that some took a proactive approach to managing these priorities. Others were more passive, trusting that things would work out and that there would be a safety net that would be adequate regardless, including food, shelter and free health care services.

#### Impact of support post-release

Personal and professional support post-release were often lifelines. When these were available, there was greater success in navigating resources and in securing shelter, food, and emotional support especially for the short term. *“I had my one friend […] him and his partner*, *they supported me*, *went out and got me some clothes and different things like toiletries and stuff*.*”*

Support from family and friends is not something that all participants received. For others, relations with family members were strained and as one participant described, *“I am kinda the bad apple of the tree*, *the black sheep of the family so I haven’t been close with relatives and family for a long time*.*”*

Professional supports included social service organizations such as a shelter or mental health organizations, social worker, case worker, nurse, doctor, and/or lawyer. The title of the professional role seemed to matter less than the willingness of the professional to engage, take the person seriously, not be judgmental, and to be willing to advocate. A particularly valuable role was that of peer support case workers who were willing to be involved intensely and with a wide variety of priorities and needs, being both practical and committed. There were several priorities that professional supports assisted with such as securing housing, personal identification, or setting up employment opportunities. While there often existed a multitude of individuals from the community providing support, there was also often one important facilitator who helped coordinate multiple items. As one participant voiced,

*“So I ended up talking to my case worker*, *she said she will help me get some funds and uh some in my pocket and uh help me to find where I can stay when I’m transitioning so she kinda helped me get to where I am here*.*”*

#### Obstacles with accessing services

There existed several challenges when it came to accessing services. These include complex processes, varying levels of cooperation from personnel and variations in resource availability/knowledge. Complicated processes involved long wait times, where participants described *“wanting to see a doctor but not wanting to sit in a hospital for five hours waiting to see a doctor*.*”* Another talked about how while waiting to receive methadone treatment *“most people say you know what fuck this and they just leave right*, *that’s how we all fall back down the fucking rabbit hole*.*”* There were also differing eligibility criteria and requirements for different programs whether that be age, personal identification requirement, intake processes, gains assessments or sobriety requirement. One participant expressed *“While you’re in jail you cannot apply for a rehab*, *a detox*, *because everyone requires a detox*.*”*

For others, getting in touch with the service whether that be having to travel to get there or needing a cellphone to make arrangements was also a major challenge. As one participant described,

“I mean you don’t have access to calling these places yourself, you literally have to go through them to make these calls, um so, I think that there should be more, like they should automatically offer it to people and like give them a list and be like these are all the places we can contact for you or help you contact so that you know”

Participants also discussed how working with organization/service personnel can also significantly vary with some individuals being very kind while others are not very knowledgeable or lack understanding. Participants also discussed the stigma that exists when reaching out for support, especially when their relationship with the support personnel has been for a short period of time. This stigma often impacted how participants went about meeting their needs. One participant who needed narcotics for their care talked about how when she met with a doctor she *“wasn’t even going to ask and then ruin that relationship and have them thinking that I am like drug seeking or something*.” When working with support personnel, the importance of self-advocacy and persistence was also echoed by many participants with many having to do *“the push on everything*.*”*

Another major concern when accessing services included the scarcity of resources. Many would go to community supports such as shelters and *“hoped for the best*.*”* One participant described how after their *“14 days had unfortunately come to an end and they [community support] were wanting to extend it but the problem was they had already promised my bed to somebody and it was the only bed that was going to be available*.*”* In addition to limited resources, there was a lack of knowledge about the supports that existed with many finding support at places that they *“stayed there like beforehand*.*”* Others emphasized the importance of having a list of resources that folks could receive upon release and mentioned that

*“if you don’t know where to go you are pretty much screwed*. *Like luckily I have been out here for long enough to know*, *I know people or I know where to go or I know where to get food or I know like how to get like you know clothes or donations or whatever like basic things that you need to get by but like if you didn’t know like they [community supports] don’t help you at all with that*. *Like they don’t even know*, *that’s the problem*.*”*

#### Influence of personal identification

We heard about PID as it relates to reintegration, including the role PID plays, experiences retaining PID and barriers to obtaining PID. For many, PID was *“super important”* and played a *“big role”* in various aspects of their lives including getting a job, bank account, credit card, cell-phone, medications, accessing healthcare, proving one’s existence, accessing welfare, driving, accessing housing, and several applications and processes. One participant talked about how *“you just ain’t shit unless you have ID because you could say you’re anyone but you don’t have the credentials*, *you can’t get a job*, *you can’t get your methadone*.*”* Others said that without it, they *“would have been screwed in like many aspects”* or were turned down by their bank because they didn’t *“have enough information or valid ID or ID to prove anything*.*”* Some even mentioned that they *“just kinda you know took it upon myself to wait until I get my health card to go and get examined*.*”* While many placed high value on their PID, others did not find much value in their ID and found ways to workaround not having it. Those who did not find much value in PID often had lower expectations and found that what was available to them was adequate or all that they deserve. The types of PID that people had including health card, drivers license, birth certificate, and Ontario ID also differed based on the priorities the individual had. Furthermore, upon release from provincial correctional facilities, individuals receive an incarceration letter that includes information such as their date of admission, date of discharge, their picture, date of birth and signatures. When asked about the role these release papers played, there were also varied response with some expressing that they “*just throwed them out”* as they didn’t understand their role and that community supports also “*couldn’t understand it*.*”* For others, they played a more important role such as helping them secure temporary housing post release. As one participant noted,

“So it was actually very hard for me to get into the shelter system given that I had no identification, um what did end up helping me a little bit was on my release papers was my picture and my date of birth”

Participants also discussed varying experiences with retaining PID. Upon being released, participants came from differing circumstances with some having access to their PID pre- and post-release, some having their PID expired during incarceration or some having their PID lost during incarceration. For a few, PID was something that they always had on them post-release but for others, especially those experiencing homelessness and/or not doing so well managing their mental health or addictions, it was easily lost, frequently fell out, or was stolen. When asked about experiences retaining PID, one participant expressed,

*“You can’t because if you’re flopping around from place to place trying to find somewhere to go […] um women steal your purse*, *junkies will all take your identification and try to use it […] I’ve kinda had people steal my identification and go absolutely nowhere because I got no credit*, *so they never return your ID*, *they always burn it or throw it away*, *you can never track it back down right*?*”*

Another described the importance of retaining their ID by talking about how they “*protect it like its fricking gold or something because it’s the only thing that proves that I exist you know*?*”* Given how easily things can get lost, another talked about how happy they were that *“the women [name] at [clinic name] took my birth certificate of me*, *photocopied it and only gave me a photocopy so I wouldn’t lose it because I’m complete waste*.*”*

For those who lost their PID or had to get it renewed given its expiry, there were several barriers that existed. Cost was a major barrier with one participant expressing how it cost *“$37 for a whole new ID and some documentations and shit and uh shit*, *I’m just barely getting started*, *the basic needs they give us through Ontario Works*, *not enough to friggin get all the groceries you want”* The magnitude of information required to obtain PID such as a guarantor, other PID, and a piece of mail with one’s address on it was also particularly frustrating for many.

*“And you don’t have an actual permanent address so you can’t–you don’t have any mail with that address on it so you know that becomes a problem […] They need permanent address or a phone or something like that*. *If you don’t*, *if don’t got any of that*, *you’re refused*.

The address requirement was especially something that many participants shared concerns with. Another mentioned that they were “*pretty much stuck with the same…the same challenges*, *like I tried to get my health card like four or five times*, *bringing in you know pieces of mail with my name on it and it was just…it was ridiculous*.*”* Other barriers that existed included long wait times, varying organizational hours, re-incarceration, and travel required to get to an ID clinic or Service Ontario location. The ease with which different types of PID were obtained also differed between participants with some finding certain PID more difficult to obtain than others.

#### Emotions and uncertainty

There was a large emotional component to being released with participants experiencing both positive and negative emotions in the midst of uncertainty of returning to community living. Upon release, while some participants were excited, appreciative of their freedom and *“happy to be out*,*”* others were overwhelmed with concerns regarding next steps, frustrations with processes, and were nervous, anxious, or experienced a combination of both positive and negative emotions.

*“I don’t know if I am kind of being selfish but people who come out of the system*, *your mind’s all wrapped up with what’s going on inside the jail or the prison*, *then you gotta come out and deal with all this*.*”*

As one participant said, *“you don’t know what you’re facing or where you’re going next*.*”* Many felt unprepared, as though they had to fulfill high expectations outlined by society, overwhelmed with the follow up that had to be completed, uncertain and wishing that someone was there to support them right away after they were released.

*“I’ve been feeling a little overwhelmed because I’ve got like a lot to follow up on […] It’s easy to just get released and you’re homeless right away so…at least I am*, *so it’s tricky to remain like you know positive and hope everything works out*. *And uh it’s a lot of weight on the shoulders to uh…to make sure I am following through with everything you know*?

Uncertainty was also due to a lack of permanent housing, with an impact on their mental state. One participant described *“I gotta be in some permanent–because if I am not in permanent*, *I–my mind’s not there*. *So I can’t*, *I can’t get my life going in order if I’m not got something permanent*.*”* Along with the uncertainty that existed, many were also frustrated when they attempted to get things done given *“people just letting you down all the time*.*”* COVID-19 further made the transition more difficult as one participant described, “*I mean*, *you had to try to fight yourself back into society and because society was so locked up*, *you suffered*.*”*

Unfamiliarity of a new city also made settling challenging. As one participant said, *“it’s much more a…strenuous*. *And your anxiety level goes up*, *your frustration of trying to get things done*.*”* Some participants who had been incarcerated for a longer time were also surprised by new rules and regulations implemented due to COVID-19 that they had to adapt to upon release.

Despite all the challenges, we often heard of the desire to give back and of personal aspirations that participants had for themselves. As one participant stated *“I can see myself being in their [support workers] shoes and then some*.*”* The desire to have a life post-incarceration filled with goals was echoed by several participants.

## Discussion

Our study identified a complex web of factors impacting individuals post-release and during their reintegration into the community. Our results are supportive of previous literature in several regards [4, 6, 8, 9, 22–24]. We found that people being released were juggling an array of priorities, some of which were imposed upon them by the justice system while others grew out of their own fears and aspirations. Complex bureaucratic and administrative processes, stigma from service providers, and emotional responses were obstacles to progress. Those receiving focused and individualized support from family and community members to meet their instrumental needs were at an advantage. Discharge planning as an approach to improving the reintegration process was generally inadequate. The reported benefit among our participants of having a single case worker helping to navigate the entire spectrum of service and support needs appears to be unique in the literature.

Something novel we explored was the role of personal identification during the reintegration process. We found the role of PID in accessing services necessary for reintegration as significant and that challenges in retaining current PID could be daunting. This was especially the case for basics of daily living such as getting a job, obtaining a bank account, and accessing housing and healthcare. Participants also described challenges with retaining PID. PID was frequently expired, stolen or lost as a result of unstable living conditions. The barriers to obtaining PID such as cost and personal documentation were often insurmountable. Other studies exploring the role of PID have echoed these same concerns [9, 13]. Our exploration of PID in the context of reintegration was unique however and is something that has not shown up in the literature before.

Our approach to this study had several strengths. Our research question remained broadly concerned with reintegration steps in general, allowing us to locate the issue of PID within a complex web of interrelated factors affecting success. This is in contrast to some studies that begin with a more narrow lens such as focus on health care access. Additionally, throughout this study, our team regularly consulted with community stakeholders who directly work with this population. By gaining their feedback through an iterative process, such as when designing interview questions, we ensured our approach was relevant, sensitive and comprehensive. Our study also contributed to local Canadian data and to our knowledge is the first exploring personal identification and reintegration in this population.

Our study had several limitations. Given the COVID-19 pandemic, interviews were conducted on the phone preventing us from picking up on body language and facial expressions. Additionally, given challenges in remotely recruiting participants and funds available, we stopped our data collection after 12 interviews. Another limitation was the limited diversity in our population. Given reallocation of prisoners during the pandemic, the majority of our participants were male and identified as White. However, results may still be used to inform future studies and draw conclusions especially for those released and impacted by factors including precarious housing, multiple incarceration experiences and/or experience with mental health and substance abuse concerns.

Several key recommendations can be made from this study. Interventions aimed to enhance the transition process can be implemented during the pre-release, during release and post-release phases. These interventions, program modifications, and policy changes must occur in collaboration with those who have lived experiences being formerly incarcerated alongside local community partners who regularly interact with this population. Based on interviews, changes can be made at a system, service organization, and individual level. At a system level, changes may involve introduction of more programs and resources pre- and post-release, more funding, legal aid and simpler processes. At a service organization level, changes may include increased service accessibility, continuous support from pre- to post- incarceration, coordination amongst multiple supports, timely support and more opportunities to prioritize mental health and addiction support due to the disproportionate burden of substance use and poor mental health among this population. At an individual level, personnel in the community may be trained to be more knowledgeable about the unique challenges that are faced by incarcerated individuals in addition to being more understanding and less stigmatizing. Furthermore, recommendations to avoid challenges with obtaining and retaining PID include helping individuals arrange for PID prior to release, providing funding for those unable to afford application fees, providing alternative options for required documentation for PID applications and enhancing PID storage options through methods such as ID Safes in the community that store people’s original PID. Through implementing these measures, individuals would be better set up for success and could avoid living in circumstances characterized by instability and uncertainty which could lead to recidivism.

Furthermore, there currently exists a scarce amount of information about personal identification as a social determinant of health and it has not yet been explored in the context of reintegration into the community post-release, especially in the Canadian context. This study effectively identifies the various different factors that play a role in the reintegration process while highlighting the importance of PID as a social determinant of health. As a result, it signifies the necessity in making changes to policies and practices in a multitude of areas that would allow marginalized populations such as those formerly incarcerated to have an improved experience post-release along with improved access to PID and support with its retention. While this study effectively provides more insight into what goes on during the post-release period, it also highlights a need for more local data with a more representative population.

## Conclusion

The period following release from incarceration is a sensitive time where individuals face challenges in attending to competing priorities. One of these priorities includes obtaining and retaining PID which appears to play an important role in countless daily tasks that can contribute to the overall well-being of an individual. To overcome the challenges that exist for those leaving incarceration, it is essential that solutions involve proactive case management that bridges the pre-release and post-release periods, simplified processes for obtaining PID, better connections to health and social services, and improved pre-release planning for community support. This study sets up the foundation for this area of study. Additional research can be conducted with a more representative sample of formerly incarcerated individuals that can be used to further modify policies and practices.

## Supporting information

S1 File Interview guide. (DOCX)

S1 Checklist COREQ (COnsolidated criteria for REporting Qualitative research) checklist. (PDF)
