# Supplementary material for: Make or break: Succeeding in transition from incarceration
Source: PLoS One. 2024 Jan 18;19(1):e0296947. doi: 10.1371/journal.pone.0296947 (PMC10795975; doi:10.1371/journal.pone.0296947)
Supplement: S1 File — (DOCX) [file pone.0296947.s001.docx]

**Interview Guide**

Interview Opening

- Are you in a comfortable environment and ready to begin the interview?
- What is your first and last name?
- What is your date of birth?
- What is your gender?
- What is your ethnicity?
- What is your phone number/email so we may contact you in the future if necessary?
- What is your address so we may mail your gift card or would you prefer for it to be emailed or picked up from Shelter Health?
- What type of facility were you released from (jail, detention center, correctional facility)?
- How many times have you been in before?
- How long were you in for the last time?
- What was the month and year of your release?

Pre-Release Period

1. Can you tell me about what was happening in the months and weeks leading up to your release period?
   1. Did you have any knowledge you were going to be released on that date?
   2. Were there any plans made with a social worker, nurse or anyone else to support you upon release?
   3. Were you worried about being released?
   4. Were there any family members or friends that were there for you upon release?
2. Did you have any identification when you went into jail such as a health card?
   1. If no, can you tell me more about that?
   2. If yes, did you still have access to it when you came out or did it expire?

Release

1. Can you tell me about what happened when you were released?
   1. Were you released from jail or from court?
   2. Based on your experience being released, did any previous plans have to change?

Post-Release

1. After you were released what did you do?
   1. Where did you stay your first night?
   2. How did you know you would stay there?
2. How have your experiences been accessing health, financial and social services upon release?
3. Was there a point where you wanted to go to a doctor but couldn't?
4. If yes, what did you do in this situation? Did you know about places that accept patients without a health card?
5. What have your experiences been with personal identification (such as birth certificate or health card) upon release?
6. What role do you feel that personal identification plays in your life?
7. Can you tell me more about your experience with obtaining personal identification?
8. Were there any challenges you faced?
9. What has your experience been like keeping your personal identification?
10. Is there anything that you think could make this process easier?

Closing

1. Is there anything else you would like to share with me about this topic that we did not have a chance to cover as yet?
